# Draft Genome Sequence of the Marine Pathogen *Vibrio coralliilyticus* RE22

**DOI:** 10.1128/genomeA.01432-15

**Published:** 2015-12-03

**Authors:** Edward Spinard, Linda Kessner, Marta Gomez-Chiarri, David C. Rowley, David R. Nelson

**Affiliations:** a Department of Cell and Molecular Biology, University of Rhode Island, Kingston, Rhode Island, USA; b Department of Fisheries, Animal and Veterinary Sciences, University of Rhode Island, Kingston, Rhode Island, USA; c Department of Biomedical and Pharmaceutical Sciences, University of Rhode Island, Kingston, Rhode Island, USA

## Abstract

*Vibrio coralliilyticus* RE22 is a causative agent of vibriosis in larval bivalves. We report here the draft genome sequence of *V. coralliilyticus* RE22 and describe additional virulence factors that may provide insight into its mechanism of pathogenicity.

## GENOME ANNOUNCEMENT

*Vibrio coralliilyticus* RE22 (formerly *Vibrio tubiashii* RE22) is a marine pathogen and a causative agent of vibriosis in larval bivalves (1). The disease is characterized by high mortality rates leading to a severe loss of production in shellfish hatcheries (2–4). Currently, only two proteases (VtpA and VtpB) and one hemolysin (VthA) have been characterized in RE22 (5–7). To better understand the mechanisms of pathogenicity, it is necessary to discover additional potential virulence factors. Here, we announce the draft genome sequence of *V. coralliilyticus* RE22 and selectively describe some potential virulence factors.

*V. coralliilyticus* RE22Sm (a spontaneous mutant resistant to streptomycin) was grown overnight in yeast-peptone broth supplemented with 3% NaCl (YP30) at 27°C in a shaking water bath. Genomic DNA was isolated using the Wizard genomic DNA purification kit (Promega), according to the manufacturer’s instructions, except DNA was resuspended into 100 µl of a 2 mM Tris-HCl (pH 8) solution. DNA was sequenced at the Rhode Island Genomics Sequencing Center, Kingston, RI, using an Illumina MiSeq Sequencer. Reads were trimmed using the CLC Genomics Workbench (version 8.0.1) for quality, ambiguous base pairs, adapters, duplicates, and size, resulting in 7,602,646 paired-end and mate-paired reads averaging 235.84 bp in size. The reads were assembled using the *de novo* assembly algorithm of CLC Genomics Workbench and SPAdes genomic assembler (version 3.1.1) (8). Contigs with an average coverage of >110 reads were joined using the CLC Microbial Genome Finishing module using *V. coralliilyticus* OCN014 as a reference genome. In total, the draft genome is composed of five contigs. Three contigs totaling 3.46 Mbp and having an average G+C content of 46% mapped to chromosome 1 of *V. coralliilyticus* OCN014. The complete chromosome 2 is represented by one 1.90-Mbp contig with a G+C content of 45%. A megaplasmid is represented by one 0.32-Mbp contig with a G+C content of 50%. The draft genome was annotated using Rapid Annotations using Subsystems Technology (RAST) and resulted in 5,234 open reading frames (9–11).

The genome of *V. coralliilyticus* RE22 encodes two extracellular metalloproteases besides those encoded by the previously described *vtpA* and *vtpB* genes. One protease shows similarity to the Epp protease in *Vibrio anguillarum* (12), while the other contains a domain conserved in the M4 family of metalloproteases (13–17). In addition to *vthA*, three putative hemolysin/cytolysin genes were discovered. A putative MARTX toxin operon encoding three type 1 secretion system (T1SS) transport proteins, a MARTX toxin, and a hypothetical protein is on the megaplasmid. Unlike typical MARTX toxin gene clusters, the transporter genes are not transcribed divergently from the MARTX toxin (18). Instead, they seem to be in the MARTX operon, upstream of the MARTX toxin gene. Unlike most MARTX toxin gene clusters, no *rtxC* (acyltransferase) is present in the operon. Additional putative hemolysins include a phospholipase/hemolysin located on chromosome 2 that shows similarity to *plp* in *V. anguillarum* (19) and a hemolysin annotated as *hlyA* located on chromosome 1 that shows similarity to *vah1* in *V. anguillarum* (20).

### Nucleotide sequence accession numbers.

This whole-genome shotgun project has been deposited in DDBJ/ENA/GenBank under the accession no. LGLS00000000. The version described in this paper is the first version, LGLS01000000.
